# Prognostic Significance of ESR1 Gene Amplification, mRNA/Protein Expression and Functional Profiles in High-Risk Early Breast Cancer: A Translational Study of the Hellenic Cooperative Oncology Group (HeCOG)

**DOI:** 10.1371/journal.pone.0070634

**Published:** 2013-07-29

**Authors:** George Pentheroudakis, Vassiliki Kotoula, Anastasia G. Eleftheraki, Eleftheria Tsolaki, Ralph M. Wirtz, Konstantine T. Kalogeras, Anna Batistatou, Mattheos Bobos, Meletios A. Dimopoulos, Eleni Timotheadou, Helen Gogas, Christos Christodoulou, Kyriaki Papadopoulou, Ioannis Efstratiou, Chrisoula D. Scopa, Irene Papaspyrou, Dimitrios Vlachodimitropoulos, Helena Linardou, Epaminontas Samantas, Dimitrios Pectasides, Nicholas Pavlidis, George Fountzilas

**Affiliations:** 1 Department of Medical Oncology, Ioannina University Hospital, Ioannina, Greece; 2 Department of Pathology, Aristotle University of Thessaloniki School of Medicine, Thessaloniki, Greece; 3 Section of Biostatistics, Hellenic Cooperative Oncology Group, Data Office, Athens, Greece; 4 Laboratory of Molecular Oncology, Hellenic Foundation for Cancer Research, Aristotle University of Thessaloniki School of Medicine, Thessaloniki, Greece; 5 Stratifyer Molecular Pathology GmbH, Cologne, Germany; 6 Department of Medical Oncology, “Papageorgiou” Hospital, Aristotle University of Thessaloniki School of Medicine, Thessaloniki, Greece; 7 Translational Research Section, Hellenic Cooperative Oncology Group, Data Office, Athens, Greece; 8 Department of Pathology, Ioannina University Hospital, Ioannina, Greece; 9 Department of Clinical Therapeutics, “Alexandra” Hospital, University of Athens School of Medicine, Athens, Greece; 10 First Department of Medicine, “Laiko” General Hospital, University of Athens, Medical School, Athens, Greece; 11 Second Department of Medical Oncology, “Metropolitan” Hospital, Piraeus, Greece; 12 Department of Pathology, Aristotle University of Thessaloniki School of Medicine, Thessaloniki, Greece; 13 Department of Pathology, University Hospital, University of Patras Medical School, Patras, Greece; 14 Department of Pathology, Alexandra Hospital, Athens, Greece; 15 Laboratory of Pathology, Evgenidion Hospital, University of Athens Medical School, Greece; 16 First Department of Medical Oncology, “Metropolitan” Hospital, Piraeus, Greece; 17 Third Department of Medical Oncology, “Agii Anargiri” Cancer Hospital, Athens, Greece; 18 Oncology Section, Second Department of Internal Medicine, “Hippokration” Hospital, Athens, Greece; University of Torino, Italy

## Abstract

**Background:**

Discrepant data have been published on the incidence and prognostic significance of ESR1 gene amplification in early breast cancer.

**Patients and Methods:**

Formalin-fixed paraffin-embedded tumor blocks were collected from women with early breast cancer participating in two HeCOG adjuvant trials. Messenger RNA was studied by quantitative PCR, ER protein expression was centrally assessed using immunohistochemistry (IHC) and ESR1 gene copy number by dual fluorescent in situ hybridization probes.

**Results:**

In a total of 1010 women with resected node-positive early breast adenocarcinoma, the tumoral ESR1/CEP6 gene ratio was suggestive of deletion in 159 (15.7%), gene gain in 551 (54.6%) and amplification in 42 cases (4.2%), with only 30 tumors (3%) harboring five or more ESR1 copies. Gene copy number ratio showed a significant, though weak correlation to mRNA and protein expression (Spearman's Rho <0.23, p = 0.01). ESR1 clusters were observed in 9.5% (57 gain, 38 amplification) of cases. In contrast to mRNA and protein expression, which were favorable prognosticators, gene copy number changes did not obtain prognostic significance. When ESR1/CEP6 gene ratio was combined with function (as defined by ER protein and mRNA expression) in a molecular classifier, the Gene Functional profile, it was functional status that impacted on prognosis. In univariate analysis, patients with functional tumors (positive ER protein expression and gene ratio normal or gain/amplification) fared better than those with non-functional tumors with ESR1 gain (HR for relapse or death 0.49–0.64, p = 0.003). Significant interactions were observed between gene gain/amplification and paclitaxel therapy (trend for DFS benefit from paclitaxel only in patients with ESR1 gain/amplification, p = 0.066) and Gene Functional profile with HER2 amplification (Gene Functional profile prognostic only in HER2-normal cases, p = 0.029).

**Conclusions:**

ESR1 gene deletion and amplification do not constitute per se prognostic markers, instead they can be classified to distinct prognostic groups according to their protein-mediated functional status.

## Introduction

Breast adenocarcinoma is the most common malignant tumor in females with 60–70% of affected patients presenting with localized disease [1]. Among predictive models, estrogen receptor (ER) protein expression, studied by means of immunohistochemical (IHC) staining, is the gold standard for the selection of patients who will be managed with hormonal therapy, carrying a weak prognostic and a moderate predictive value for benefit from such treatment [2], [3]. The advent of robust, sensitive and reproducible reverse-transcriptase polymerase chain reaction (RT-PCR) techniques analyzing messenger RNA (mRNA) reliably quantify expression of genes and provide normalized ER gene expression data [4], [5]. Still, the prognostic/predictive value of tumoral ER gene expression and its correlation to protein expression and gene copy number aberrations have not been thoroughly studied to date.

Gene amplification of the ESR1 gene, encoding the ER, has been the focus of recently published studies, as gene amplification is the major mechanism behind the cancer-related changes of many oncogenes, including ERBB2 (HER2) [6]–[11]. These studies reported discrepant results and generated much debate about the frequency of ESR1 amplification, its association to clinicopathologic tumor charasteristics and its prognostic significance. Moreover, contradictory data showed ESR1 gene amplification to be associated with sensitivity and, in other publications, with resistance to tamoxifen [6]–[11].

Consequently, we took advantage of the «trial quality» collection of well annotated formalin-fixed paraffin-embedded (FFPE) tumor blocks from early breast cancer patients randomized in two prospective clinical trials of the Hellenic Cooperative Oncology Group (HeCOG) in order to globally profile ESR1 gene copy number aberrations, mRNA and protein expression and study their incidence, correlations, prognostic and predictive utility [12], [13]. We also intended to investigate the prognostic significance of complex molecular phenotypes that reflect ESR1 structural and functional status.

## Patients and Methods

**Figure 1:**
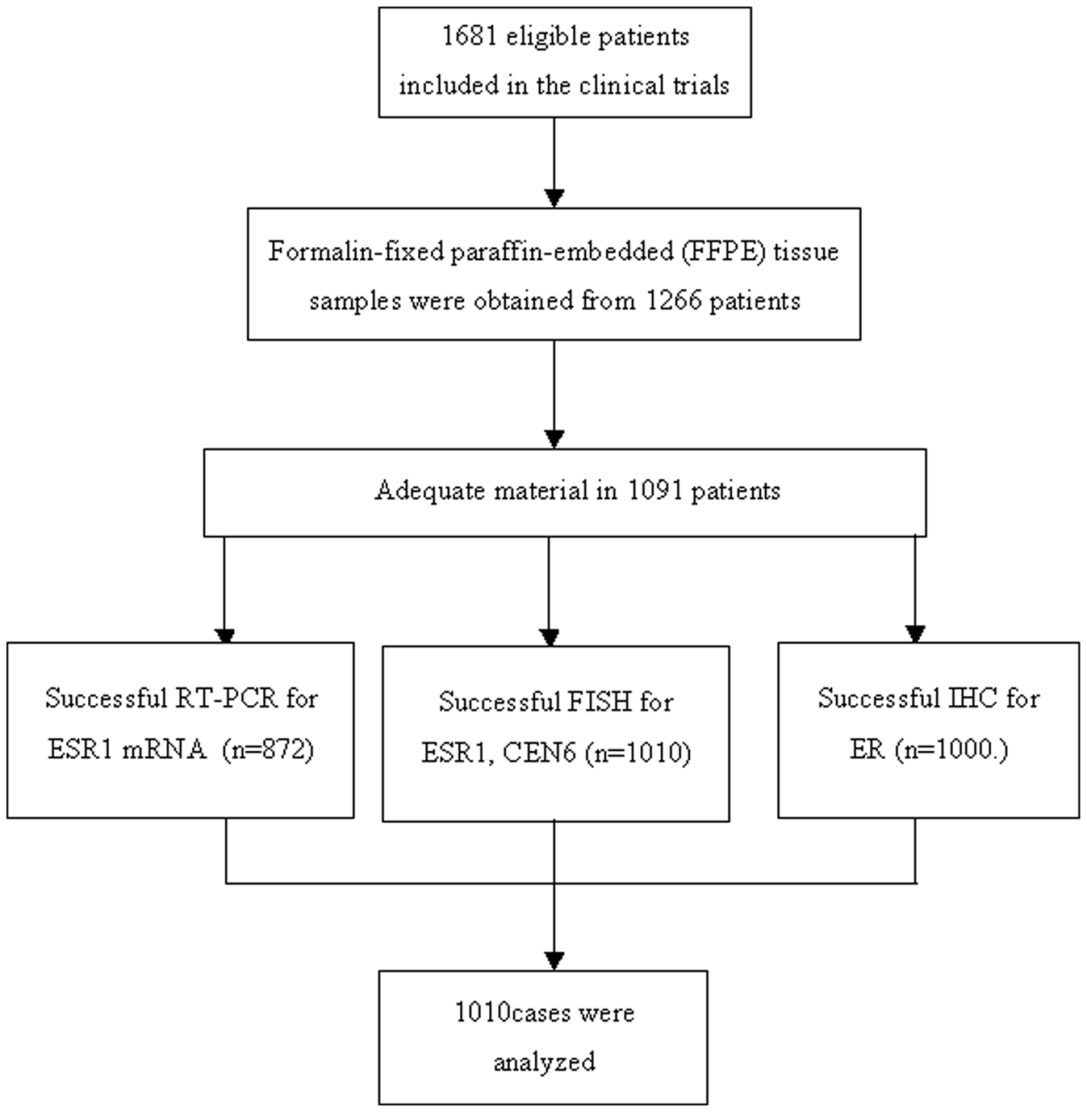
REMARK flow chart.

This was a retrospective translational research study amongst patients who had been enrolled in two prospective clinical trials (A REMARK diagram is provided in Fig. 1). The HeCOG prospective trial HE10/97 randomised a total of 595 high-risk (T1-3N1M0 or T3N0M0) breast cancer patients to either four cycles of epirubicin followed by four cycles of intensified cyclophosphamide, methotrexate and 5-fluorouracil (E-CMF) or three cycles of epirubicin followed by three cycles of paclitaxel and three cycles of intensified CMF (E-T-CMF) every two weeks [12]. The prospective trial HE 10/10 randomized a similar population of 1121 node-positive, early breast cancer patients to the prior E-T-CMF or a ET-CMF arm [13]. Clinical protocols were approved by local regulatory authorities and were also included in the Australian New Zealand Clinical Trials Registry (ANZCTR) and allocated the following Registration Numbers: ACTRN- 12611000506998 (HE10/97) and ACTRN12609001036202 (HE10/00). The translational research protocol was approved by the Bioethics Committee of the Aristotle University of Thessaloniki School of Medicine (A7150/18-3-2008). All patients signed a study-specific written informed consent before randomization.

### IHC

Collection of formalin-fixed paraffin-embedded (FFPE) tumor tissue samples was possible in 1010 patients (Fig. 1), evaluated histologically and recorded for the percentage of tumor cell content. Immunohistochemical staining was performed according to standard protocols, with slight modifications, on serial 2.5-µm-thick sections from Tissue Microarray (TMA) blocks, constructed with the use of a manual arrayer (Model I, Beecher Instruments, Sun Prairie, WI, USA), using two cores per case of 1.5 mm in diameter. ER IHC (clone 6F11, Leica Biosystems, Newcastle Upon Tyne, UK, dilution 1∶70) was processed and evaluated at the Laboratory of Molecular Oncology of the Hellenic Foundation for Cancer Research, Aristotle University of Thessaloniki School of Medicine. Other antibodies (HER2, Ki67) were processed according to standardised protocols, as published elsewhere [12]. ER tumor staining was assessed by means of three different scoring methods: a) the percentage of tumor cells with stained nuclei, b) the Allred score, c) the semiquantitative H-Score [H-score = (1×percentage of weakly positive cells)+(2×percentage of moderately strong positive cells)+(3×percentage of strongly positive cells).range 0–300), range 0–300 [13]. Cut-offs for categorization to ER-positive or negative cases were a) at least 1% of malignant cells with stained nuclei, b) Allred Score >2, c) H-score >50. For Ki67, the expression was defined as low (<14%) or high (≥14%) based on the percentage of stained/unstained nuclei from the tumor areas [14]. HER2 protein expression was scored in a scale from 0 to 3+, the latter corresponding to uniform, intense membrane staining in >30% invasive tumor cells [15].

### RT-PCR

Prior to RNA isolation, macrodissection of tumor areas was performed in most of the FFPE sections with <50% tumor cell content. RNA was isolated using a standardized fully automated isolation method for total RNA from FFPE tissue, based on silicagermanium-coated magnetic beads (XTRAKT RNA kits, STRATIFYER Molecular Pathology GmbH, Cologne, Germany) in combination with a the liquid handling robot XTRAKT XL (STRATIFYER Molecular Pathology GmbH, Cologne, Germany) . The method involves extraction-integrated deparaffinization and DNase I digestion steps. . The quality and quantity of RNA was checked by measuring CALM2 expression as a surrogate for amplifiable mRNA by qRT-PCR. CALM2 was used as endogenous reference, since it had previously been identified as stably expressed among breast cancer tissue samples.

Expression of the target gene, as well as the reference gene CALM2, was assessed in triplicate by qRT-PCR using the SuperScript III PLATINUM One-Step Quantitative RT-PCR System with ROX (Invitrogen, Karlsruhe, Germany) in a Stratagene Mx3005p (Agilent Technologies, Böblingen, Germany). The lengths of the amplicons detected by the ESR1 and CALM2 assays were 73 bp and 72 bp, respectively, with PCR efficiencies [E = 1(10-slope)] of 101.0% and 99.70%, respectively. Forty cycles of nucleic acid amplification were applied and the cycle threshold (CT) values of the target gene were identified. CT values were normalized by subtracting the CT value of the housekeeping gene CALM2 from the CT value of the target gene (ΔCT). RNA results were then reported as 40-ΔCT values, which correlate proportionally to the mRNA expression level of the target gene.A commercially available human reference RNA (Stratagene qPCR Human Reference Total RNA, Agilent Technologies, Waldbronn, Germany) was used as positive control.

The Primer/Probe (FAM/TAMRA-labeled) sets used for amplification of the target and reference genes were the following (5′→3′):

ESR1 (NM_000125)

Probe ATGCCCTTTTGCCGATGCA

Forward Primer GCCAAATTGTGTTTGATGGATTAA

Reverse Primer GACAAAACCGAGTCACATCAGTAATAG

CALM2 (NM_001743)

Probe TCGCGTCTCGGAAACCGGTAGC

Forward Primer GAGCGAGCTGAGTGGTTGTG

Reverse Primer AGTCAGTTGGTCAGCCATGCT

### FISH

**Figure 2:**
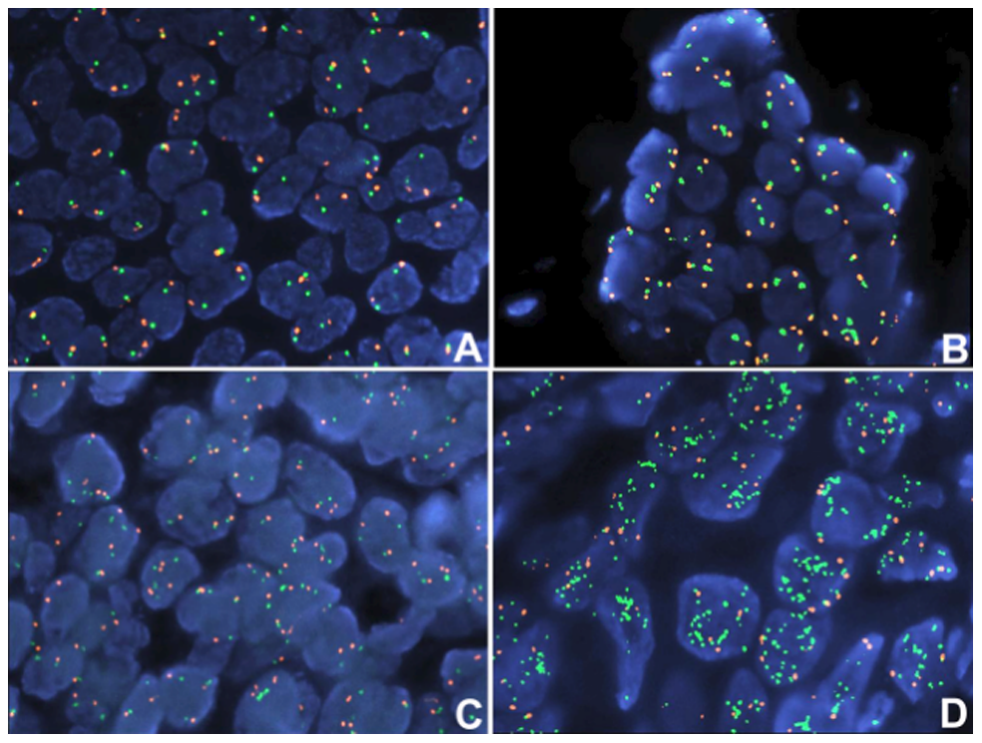
Fluorescence in situ hybridization (FISH) in invasive breast carcinomas (IBC) using the ESR1/CEP6 dual color probe. ESR1 gene (green signals) in an IBC case with normal gene status is presented (**A**), IBC cases with gain of ESR1 gene (**B–C**) and in the last panel (**D**), case with high amplification of ESR1 gene, accompanied by gain of CEP6. Magnification ×1000. CEP6, centromere 6 enumeration probe.

TMA sections (5 µm thick) were cut for FISH analysis, using the ZytoLight® SPEC ESR1/centromere 6 (CEP6) dual color probe kit and the ZytoLight® SPEC HER2/TOP2A/centromere 17 (CEP17) triple color probe kit (both from ZytoVision, Bremerhaven, Germany). FISH was performed according to the manufacturer's protocol with minor modifications. For all probes, sequential digital images were captured by a stack motor (5 planes at 1.0 µm for each probe) using the Plan Apo VC 100×/1.40 oil objective (Nikon, Japan) using specific filters and the resulting images were reconstructed with the appropriate pseudo-colors using the XCyto-Gen software (ALPHELYS, Plaisir, France). For HER2/CEP17 status a minimum of 20 tumor cells were counted, whereas for the ESR1/CEP6 status, 40 to 60 cells 23 [16]. The HER2 gene was considered to be amplified when the ratio of the respective gene probe/centromere probe was >2.2 or the HER2 copy number was >6 [17]. The cases were scored as ESR1 deleted when the ratio gene/CEP was <0.8, normal between ≥0.8–≤1.0, gene gain >1.0–<2.0, and amplified if the ratio was ≥2.0 or the gene copy number >6 [6], [7], [18], [19]. ESR1 gene enumeration was performed using counting guides for other genes (HER2, TOP2A) with minor changes, as well as the probe manufacturer's recommendations. The size of the ESR1 signals of the surrounding normal cells was used to decide whether the ESR1 signal size was enlarged. In clusters, the number of ESR1 signals was estimated based on the diameter of the gene signal found in normal breast epithelium (Figure 2). The observers performed FISH analyses blinded to the results of the IHC and PCR assays.

### Statistical analysis

Disease-free survival (DFS) was measured from the date of randomization until recurrence of tumor or secondary neoplasm or death from any cause. Overall survival (OS) was measured from the date of randomization until death from any cause. Time-to-event distributions were estimated using Kaplan–Meier curves and comparisons were made using log-rank test [17]. Univariate Cox regression analyses, adjusted for paclitaxel treatment, were performed to assess the prognostic significance of markers with DFS or OS. Interaction tests of the examined markers with paclitaxel treatment, menopausal status, hormonal therapy and HER2 status were performed as exploratory analyses with level of significance α = 10%. In the multivariate Cox regression analysis a backward selection procedure based on likelihood ratio with a removal criterion of p>0.10 was applied. Clinicopathological parameters such as treatment group (paclitaxel vs. no-paclitaxel treatment), menopausal status, involved axillary lymph nodes (>4 vs. 0–3), histological grade (III–Undifferentiated vs. I–II), tumor size (>5 and 2–5 vs. <2), adjuvant hormonal therapy, histology, Ki67 protein expression and HER2 status were entered in the initial step of the model. In multivariate analysis, we included the complex ESR1 Gene Functional profile (see section *Complex molecular profiles: Gene Functional classification*) along with the standard clinicopathologic characteristics cited above. Final models were presented using forest plots. Results of this study are reported as per the corresponding recommendations for tumor marker prognostic studies [18] .

## Results

### Patient and Tumor Demographics

A total of 1010 women with resected early breast adenocarcinoma, mostly >T1 (68.7%), node-positive (99.6%, N2 in 60%) and ER-positive (77%) were managed with anthracycline and taxane-based chemotherapy (84.2%) and hormonal therapy (78.3%). Only 159 patients (15.9%) did not receive paclitaxel. Basic patient and tumor characteristics are summarized in Table 1. There were no significant differences between patient and tumor characteristics of the two trials with those of our study cohort.

**Table 1:** Patient and Tumor Demographics.

Patient and Tumor Demographics	N = 1010
*Median age (range)*	52.5 (22.4–79.3)

	N (%)
*Randomization group*	
E-T-CMF	486 (48.1)
E-CMF	159 (15.7)
ET-CMF	365 (36.1)
*Menopausal status*	
Premenopausal	471 (46.6)
Postmenopausal	539 (53.4)
*Tumor size (cm)*	
≤2	316 (31.3)
2–5	569 (56.3)
>5	125 (12.4)
*Number of positive axillary lymph nodes*	
0–3/≥4	400 (39.6)/610 (60.4)
*Tumor grade*	
I–II/III–IV	499 (49.4)/511 (50.6)
*Histology classification*	
Invasive ductal	788 (78.0)
Invasive lobular	100 (9.9)
Mixed	73 (7.2)
Other	49 (4.9)
*Estrogen Receptor Status*	
Negative/Positive	227 (22.5)/778 (77.0)
*HER2 IHC3+ and/or FISH+*	247 (24.5)
*Ki67 (n = 987)*	
Low (<14%)	312 (31.6)
High (>14%)	675 (68.4)
*Hormonal therapy*	791 (78.3)
*Tamoxifen/Aromatase inhibitors*	696 (68.9)/153 (15.1)

At a median follow-up of 105.5 months, 303 (30%) experienced tumor relapse and 262 (25.9%) had died. The 5-year DFS and OS rates were 73.6% (70.9–76.3) and 86.5% (84.3–88.6) respectively. No statistically significant DFS or OS survival difference was seen between E-T-CMF, E-CMF, ET-CMF in the HeCOG trials (data published) nor in our patient cohort under study (data not shown) [12], [13].

### Study biomarkers and correlations

503 tumors (49.8%) had more than two ESR1 gene copies while only 30 (3%) of them had five or more copies. On the other hand, 499 (49.4%) tumors had less than two ESR1 gene copies. The tumoral ESR1/CEP6 ratio was suggestive of deletion (<0.8) in 159 (15.7%), normal status (0.8–1) in 258 (25.5%), gene gain (>1 to <2) in 551 (54.6%) and amplification (ratio≥2) only in 42 cases (4.2%). Gene clusters were observed in 96 cases (9.5%) and were scored as gene ratio normal in one, gain in 57 and amplification in 38. Of note, 38 out of the 42 ESR1-amplified cases (90.5%) were shown tight gene cluster .

The 40-ΔCt mRNA value distribution of ESR1 had a median value of 40.5 (range 28.5–46) and 654 tumours had ESR1 mRNA values higher than the 25th distribution percentile. Among the majority of ER-positive breast carcinomas (77%), strong ER protein expression as defined by Allred score>6 or H score>200 was noted in 10–15% of tumors (Table 2).

**Table 2:** Distribution of study biomarkers.

	N (%)	Median (range)
**FISH markers**		
*ESR1 gene copies (n = 1010)*		2.0 (0.55–16.13)
<2	507 (50.2)	
≥2–<5	473 (46.8)	
≥5	30 (3.0)	
*CEP6 gene copies (n = 1010)*		2.0 (0.55–6.77)
<2	510 (50.5)	
≥2–<5	494 (48.9)	
≥5	6 (0.6)	
*ESR1/CEP6 ratio (n = 1010)*		1.03 (0.35–6.74)
Deletion (ratio<0.8)	159 (15.7)	
Normal (0.8–1)	258 (25.5)	
Gain (1<ratio<2)	551 (54.6)	
Amplified (ratio≥2)	42 (4.2)	
**mRNA markers**		
*ESR1 (ΔCT values) (n = 872)*		40.53 (28.52–46.00)
Low (<25th percentile)	218 (25.0)	
High (>25th percentile)	654 (75.0)	
**IHC markers**		
*ER status (n = 1000)*		
Negative (0)	263 (26.3)	
Positive (≥1%)	737 (73.7)	
*ER Allred score*		
0–2 (negative)	266 (26.6)	
3–6	627 (62.7)	
7–8	107 (10.7)	
*ER H-score*		
<50	344 (34.1)	
50–200	506 (50.1)	
>200	150 (15.0)	

**Figure 3:**
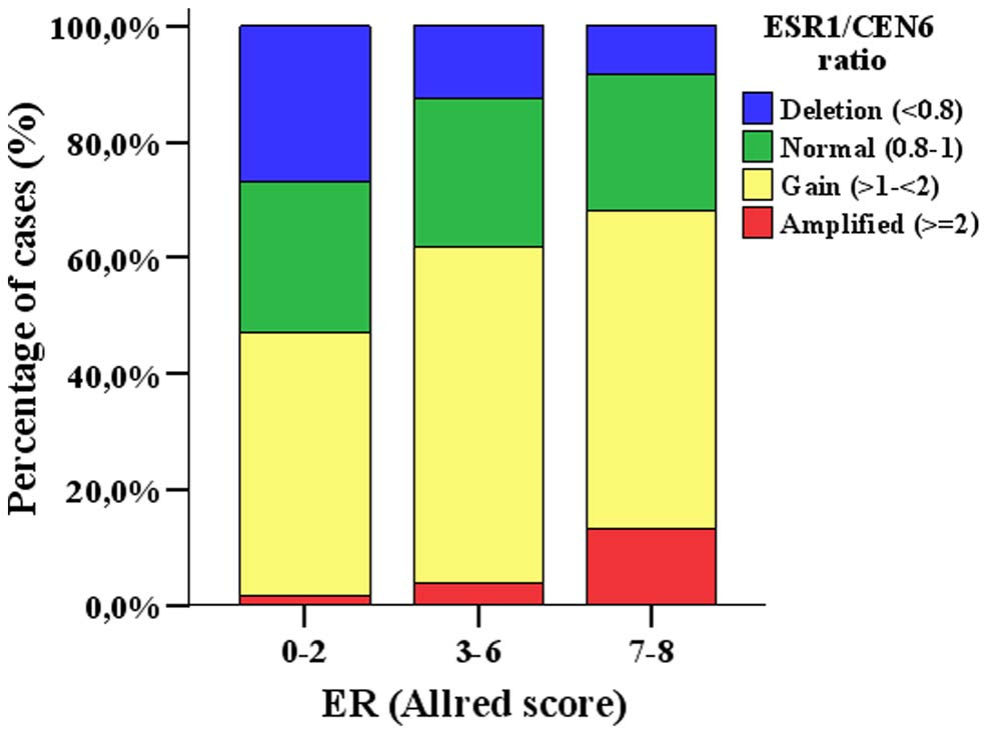
Bar chart of ER IHC protein expression by ESR1/CEP6 gene ratio.

When examined as categorical variables, the ESR1/CEP6 gene ratio was significantly associated with ER protein expression by any scoring algorithm as well as with ESR1 mRNA expression (p<0.001, Supplementary Table S1). ESR1 gene gain and amplification were associated with positive protein and mRNA expression. Moreover, positive ESR1 mRNA expression was significantly associated with positive ER protein expression (p<0.001). However, when we examined the correlations of continuous variables such as ESR1 40-ΔCt mRNA, ESR1 gene copy number or ratio values, ER IHC protein expression (H score or Allred score), evidence for robust correlation was only seen between ESR1 mRNA with protein expression (Spearman's Rho 0.66, p<0.0005). The correlation of ESR1 copy number or ESR1/CEP6 ratio with either mRNA values (Spearman's Rho 0.085–0.22, p = 0.01<0.012) or protein H score (Spearman's Rho 0.098–0.23, p = 0.002<0.002) was statistically significant but rather weak, especially for complex biological systems. This observation supports the presence of ESR1 gene gain/amplification events that did not translate in increased synthesis of the relevant protein as well as presence of cases with strong ER protein expression which was due to mechanisms other than gene gain/amplification. Figure 3 shows the distribution of tumor ER protein expression by Allred score in various ESR1/CEP6 ratio categories. Eighty-seven breast cancers with ESR1 gene deletion showed moderate and strong ER protein expression, while 121 cases with ESR1 gene gain had no ER protein expression. Among the rare breast tumors with ESR1 gene amplification, 24 harbored moderate and 14 strong ER protein expression but four showed none. Among the 96 cases with ESR1 clusters, 11 showed no ER protein and five low ER mRNA expression.

Similarly, 80 breast tumors with gene deletion contained mRNA levels higher than the 25th percentile, whereas 97 tumors with gene gain and five with gene amplification were found to have ESR1 mRNA levels lower than the cut-off used. . No significant association of ESR1/CEP6 gene ratio with HER2 amplification/overexpression or Topoisomerase-IIA gene amplification was seen.

We examined the association of ESR1/CEP6 gene ratio, mRNA and protein expression with standard clinicopathological characteristics. Both positive ER protein expression (by % staining cells, Allred and H scores) as well as positive mRNA expression were significantly associated with histological grade I/II and invasive lobular histology (Fisher's exact p = 0.001). Increasing ESR1 gene copy number was significantly correlated with age>50 (p = 0.001), postmenopausal status (p = 0.001), high histological grade III–IV (p = 0.017) and invasive ductal histology (p = 0.009). Gene gain and amplification by ESR1/CEP6 ratio was significantly associated with age>50 (p = 0.003), postmenopausal status (p = 0.009) and high-grade histology (grade III–IV) (p = 0.022).

### Prognostic significance of study biomarkers

Tumor ER protein expression by any scoring algorithm was associated with favorable patient outcome. Patients harboring tumors with >1% ER-staining cells had a Hazard Ratio (HR) of 0.72 for disease relapse and 0.67 for death, as shown in Table 3. In fact when the Allred and H scores were analyzed, the HR for malignant relapse ranged from 0.72 to 0.82 for cases with weak/moderate expression and from 0.65 to 0.66 for cases with strong protein expression. Similarly, the HR for risk of death was 0.66–0.75 for cases with weak/moderate ER protein expression and 0.57–0.62 for tumors with strong IHC staining. A significant interaction between menopausal status and ER protein expression in terms of DFS was found (Wald's p = 0.012). More specifically, in premenopausal patients positive ER tumors (Allred score 3–8) were associated with lower risk for relapse (HR = 0.523, 95% CI: 0.377–0.724, Wald's p<0.001) compared to negative ER tumors (Allred score 0–2). In postmenopausal patients no significant difference was found (HR = 0.933, 95% CI: 0.683–1.275, Wald's p = 0.663). In terms of OS the interaction between the two parameters was not significant (Wald's p = 0.277). No significant interaction was found of ER IHC expression markers with paclitaxel treatment for either DFS or OS ( p-value>0.05 in all cases).

**Table 3:** Prognostic significance of study biomarkers in univariate analysis.

	DFS			OS		
	HR	95% CI	Wald's p	HR	95% CI	Wald's p
*ER status*						
Negative (0)	1			1		
Positive (≥1%)	0.72	0.58–0.91	**0.005**	0.67	0.51–0.87	**0.002**
*ER Allred score*						
0–2	1		**0.013**	1		**0.006**
3–6	0.72	0.58–0.91	**0.006**	0.66	0.51–0.86	**0.002**
7–8	0.66	0.45–0.98	**0.036**	0.62	0.40–0.98	**0.040**
*ER H score*						
<50	1		**0.030**	1		**0.011**
50–200	0.82	0.65–1.02	0.072	0.75	0.58–0.97	**0.028**
≥200	0.65	0.46–0.92	**0.013**	0.57	0.38–0.86	**0.007**
*ESR1 (gene copies)*						
≤2	1		0.79	1		0.089
2–5	1.03	0.83–1.27	0.80	1.15	0.89–1.47	0.28
≥5	1.22	0.68–2.20	0.50	1.89	1.04–3.43	**0.036**
*ESR1 gene status*						
Deletion	1		0.39	1		0.37
Normal	0.80	0.57–1.12	0.20	0.72	0.49–1.06	0.099
Gain	0.96	0.72–1.29	0.80	0.89	0.64–1.24	0.50
Amplified	0.73	0.39–1.35	0.31	0.76	0.38–1.51	0.43
*ESR1 mRNA expression*						
Low (<25^th^ percentile)	1			1		
High (≥25^th^ percentile)	0.90	0.70–1.16	0.43	0.74	0.56–0.99	**0.040**
*Gene Functional profile (N = 864)*						
Ratio gain, no function	1		**0.006**	1		**0.003**
Ratio normal, no function	0.78	0.52–1.15	0.21	0.86	0.55–1.35	0.52
Ratio normal, functional	0.54	0.38–0.78	**0.001**	0.49	0.32–0.75	**0.001**
Ratio gain, functional	0.64	0.46–0.88	**0.006**	0.61	0.42–0.89	**0.009**

The number of ESR1 gene copies was not prognostic for DFS, although it did predict for adverse OS. Patients with tumors harboring >5 ESR1 gene copies had a risk of death increased by 89% compared to patients with up to 2 gene copies (p = 0.036). The number of CEP6 gene copies had no prognostic significance for either DFS or OS. Similarly, the tumoral ESR1/CEP6 gene ratio showed no evidence for prognostic impact on DFS or OS. Moreover, the presence or absence of ESR1 clusters did not have prognostic utility. However, a significant interaction between ESR1/CEP6 gene ratio and paclitaxel treatment was observed for DFS (Wald's p = 0.017) and marginally for OS (Wald's p = 0.062). More specifically, in the subgroup of patients with tumoral ESR1/CEP6 gene ratio ≤1, paclitaxel treatment was non-significantly associated with increased risk of relapse (HR = 1.42, 95% CI = 0.82–2.48) and death (HR = 1.21, 95% CI = 0.66–2.23). In the subgroup of patients with gene gain or amplification (ESR1/CEP6>1), paclitaxel treatment was associated with decreased risk of relapse (HR = 0.66, 95% CI = 0.49–0.90) and death (HR = 0.63, 95% CI = 0.44–0.89). Digital FISH images can be seen at http://hecog-images.gr/ESR1/FISH_HE10/97_HE10/00

High tumor ESR1 mRNA expression significantly correlated with improved patient survival (HR for OS 0.74, p = 0.04), though not with superior DFS. No significant interaction of ESR1 mRNA expression with paclitaxel treatment was found for either DFS or OS (p-value>0.05 in all cases).

### Complex molecular profiles: Gene Functional classification

**Figure 4:**
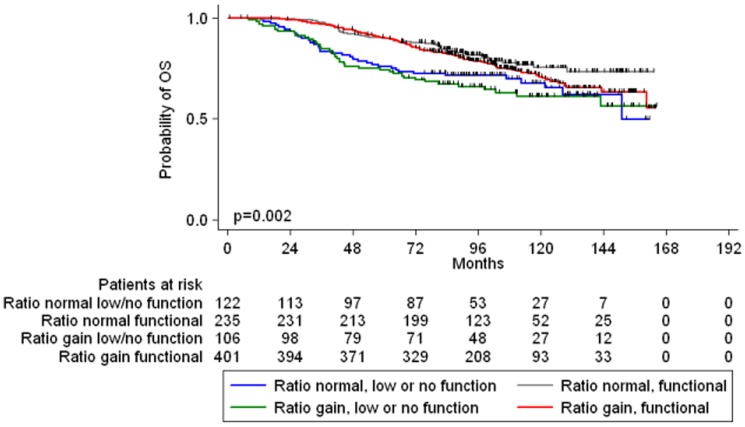
Overall Survival of patients by Gene Functional profile.

We sought to construct a molecular classifier incorporating all ESR1 study parameters with function as the main criterion. The ESR1/CEP6 gene ratio was used in order to assign tumors to the Gene Ratio Normal (Ratio ≤1) or Gene Ratio Gain (Ratio>1) feature, while ESR1 mRNA and ESR1 Allred score were used in order to assign tumors to the Functional or No Function feature. At our initial attempt, tumors were classified in six groups according to a Gene Functional profile: Gene Ratio normal, No function or functional (two groups), Gene Ratio gain, No function or Functional (two groups) and Unclassified Group 1 (Ratio normal, only one of ESR1 mRNA, ER protein expression positive), Unclassified Group 2 (Ratio gain, only one of ESR1 mRNA, ER protein expression positive). The plethora of tumor groups impacted negatively on the probability of identifying distinct prognostic cohorts and the two unclassified groups did not contribute to a biologically meaningful classification. Since proteins are ultimately the mediators of cellular function, we recoded the two unclassified groups according to protein IHC expression (Allred 0–2: no or low function, Allred 3–8: functional). Consequently, 864 breast carcinomas were classified in four groups: a) Gene Ratio normal, No function 122 (14.1%), b) Gene Ratio normal, Functional 235 (27.2%), c) Gene Ratio gain, No function 106 (12.3%), d) Gene Ratio gain, Functional 401 (46.4%). Both DFS and OS were superior for patients with Functional tumors irrespective of Gene Ratio status compared to those of non-functional tumors with ESR1 gene gain. The patient group of Gene Ratio Gain/No function had the worse DFS (p = 0.006) and OS (p = 0.002) of all four groups (Figure 4, OS). Using the poor prognosis Gene Ratio Gain/No function as the reference group, the Hazard Ratios for risk of relapse (DFS) were: Gene Ratio normal/No function 0.78, Gene Ratio normal/Functional 0.54, Gene Ratio gain/Functional 0.64. Similarly, the Hazard Ratios for risk of death (OS) were: Gene Ratio normal/No function 0.86, Gene Ratio normal/Functional 0.49, Gene Ratio gain/Functional 0.61 (Table 3).

A significant interaction between the Gene Functional profile and HER2 status (FISH amplification and/or IHC 3+ overexpression) was observed for OS (Wald's p = 0.047) but not for DFS (Wald's p = 0.14). The prognostic impact of the Gene Functional profile persisted only in patients with HER2 negative disease, but vanished in HER2 positive tumors. Moreover, a significant interaction between the Gene Functional profile and paclitaxel therapy was observed for DFS (Wald's p = 0.041) but not for OS (Wald's p = 0.17). Specifically, in tumors with normal Gene Ratio (irrespective of functional status) paclitaxel therapy was not associated with DFS benefit. On the contrary, in tumors with Gene Ratio gain the administration of adjuvant paclitaxel was marginally associated with superior DFS, irrespective of functional status.

### Multivariate Analysis

**Figure 5:**
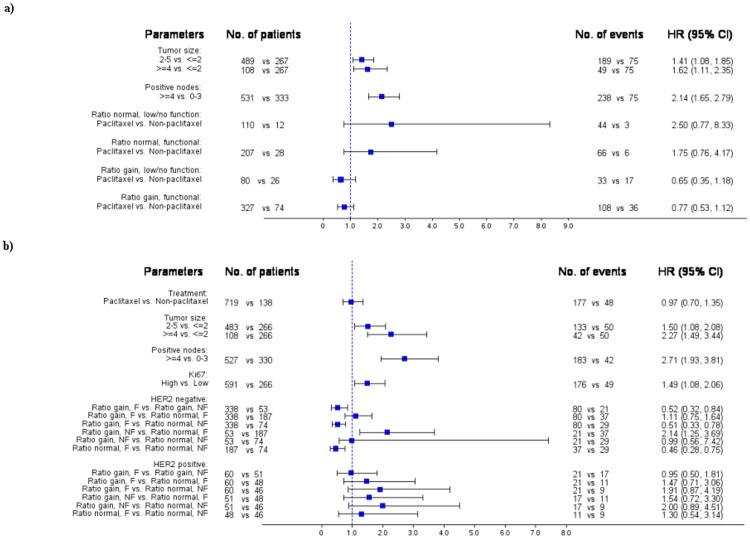
Multivariate analysis for DFS (a) and OS (b) presented by forest plots.

Forest plots in Figure 5 present multivariate analysis. . The interaction of the Gene Functional profile with paclitaxel therapy showed marginal independent significance for DFS (p = 0.066). Paclitaxel therapy was non-significantly associated with superior DFS in cases with gene gain or amplification and with inferior DFS in the absence of ESR1 gene gain. Prognostic factors with independent significance for superior OS were small tumor size, less than four involved axillary nodes, Ki67<14%, and the interaction of the Gene Functional profile with HER2 tumor status (p = 0.029). Irrespective of gene ratio status, patients with ESR1 functional tumors fared better than those with non-functional tumors only in the absence of HER2 amplification/overexpression. In the presence of HER2 amplification/overexpression, the prognostic impact of functional ESR1 was lost.

## Discussion

ER is encoded by the ESR1 gene localized on chromosome 6q25.1, and copy number changes of ESR1 have only recently become the focus of interest. Holst et al reported a FISH ESR1 amplification rate of 20.6% in 2000 breast carcinomas loaded in tissue microarrays, the majority showing a clustered arrangement of tight signals and corresponding to 12–26 gene copies per nucleus by qPCR [6]. However, other groups soon refuted these findings, reporting amplification rates as low as 0.9% [8]–[11]. Differences in patient populations, tumor characteristics and methodologies and definitions used (qPCR, MLPA, aCGH, FISH) only partly explain such discrepancies. We used strict protocol-quality guidelines for data capture and central FISH/IHC assessment in >1000 tumors in order to report an amplification rate of 4.2%, mostly low-level (five or more gene copies per nucleus in only 3% of cases) and a deletion rate of 15.7%. Our reported incidence of ESR1 amplification is intermediary between that reported by Brown (FISH, 1%) [8], Vincent-Salomon (aCGH, 0.9%) [10], Moelans (MLPA, 2%) [19], Horlings (aCGH and FISH, 2.3%) [9], Reis-Filho (FISH, 4%) [11] and that reported by Ooi (RNAse FISH, 5.9%) [18], Ejlertsen (FISH, 13.6%) [19], Nielsen (FISH, 14%) [20], Tomita (FISH, 22.6%) [7]. In contrast to Holst et al, we used a manual scoring algorithm in order to count the number of gene signals and assess the ESR1/CEP6 ratio, rather than consider all cases with tight clusters as amplification events. Cases with gene clusters were seen in 9.5% of cases (almost all scored as gain and amplification events).

Despite varying incidence, some of our findings confirm those reported by other groups. ESR1 gene amplification was low-level and correlated with high histological grade, in keeping with data reported by Ejlertsen et al [19] and Moelans et al [22]. The correlation of ESR1 gene gain or amplification with protein expression was rather weak, , in agreement with data from other groups. We report deleted ESR1 cases in 15.7%, an incidence which is higher than the one reported by Ejlertsen (4.2%) [19], though in agreement with preclinical observations showing gene deletion in four out of six breast cancer cell line [21]. Moreover, some of the deleted cases were due to a high number of CEP6 copies in the presence of normal ESR1 gene copy number. . We did observe a favorable prognostic significance of ER mRNA and protein expression, but failed to find any for ESR1 gene ratio, despite the numerical association of copy number with increased risk of relapse and death. Even when we ommitted the CEP6 gene copy number as a possible confounder and studied only ESR1 gene copies, we failed to demonstrate an unequivocal prognostic impact on DFS and OS in uni- and multivariate analysis. In contrast to the initially reported Holst data, several groups (Nielsen et al, Ejlertsen et al) [19], [23] established an adverse prognostic significance of ESR1 copy number aberrations which have been linked to tamoxifen resistance, while others failed to find any [8]–[11].

Ooi et al interpreted the decline of observed rate of ESR1 gene amplification after RNAse pretreatment as evidence suggesting that some of the gene signals identified by FISH are newly synthesized nascent RNA extending from the gene [18]. However, Moelans et al subsequently reported that although RNAse removed cloudy clusters, it did not change copy number in 12/15 amplification and in 8/9 gain events [22], [23]. Regarding ESR1 gene copy number aberrations, we consider their correlation to high histological grade, their weak association with protein expression and the discrepant incidence rates and prognostic significance reported so far as evidence suggesting that they make up a heterogeneous group of genomic abnormalities. This broad group includes gene gain/amplification cases with no structural or regulatory abnormalities that result in increased protein expression as well as gain/amplification cases in which the ESR1 gene, abnormal in structure or copy number, fails to regulate other genes or to translate to ER protein. Indeed, when we combined gene status, mRNA and protein expression in a single molecular classifier, the functional status of each case was the only significant predictor of outcome both in univariate and multivariate analysis, irrespective of the gene copy number. Of interest, an unplanned, exploratory analysis suggested that the gene copy number gain/amplification retained predictive significance for paclitaxel benefit, a finding warranting validation in an independent cohort. The prognostic significance of gene functional groups only persisted in breast carcinomas without HER2 amplification/overexpression. Similarly, Ejlertsen et al reported an adverse prognostic role of ESR1 gene amplification only in HER2-normal cases [19]. It is likely that the major effects of HER2 gene activation on cellular function make the impact of ESR1 gene copy number/function status irrelevant.

In conclusion, our data confirm the prognostic (or predictive) significance of ER mRNA and protein expression in high-risk early breast cancer and highlight the heterogeneous nature of ESR1 gene copy number aberrations with respect to regulatory and functional impact on the cancer cell. ESR1 gene deletion and amplification do not constitute per se prognostic markers, instead they can be classified to distinct prognostic groups according to their protein-mediated functional status. Further research is warranted on the prognostic differences of these functional groups according to gene copy number changes and on the correlation of ESR1 gene copy number to paclitaxel benefit and HER2 signalling.

## Supporting Information

Table S1 Association of ESR1/CEP6 gene ratio with mRNA and protein expression. (DOC)
