# Supplementary material for: Prognostic Significance of ESR1 Gene Amplification, mRNA/Protein Expression and Functional Profiles in High-Risk Early Breast Cancer: A Translational Study of the Hellenic Cooperative Oncology Group (HeCOG)
Source: PLoS One. 2013 Jul 29;8(7):e70634. doi: 10.1371/journal.pone.0070634 (PMC3726626; doi:10.1371/journal.pone.0070634)
Supplement: Table S1 — (DOC) [file pone.0070634.s001.doc]

|  | **ESR1/CEP6 gene ratio** | | | |  |
| --- | --- | --- | --- | --- | --- |
|  | Deletion | Normal | Gain | Amplified | p |
|  | **N (%)** | **N (%)** | **N (%)** | **N (%)** |  |
| **IHC** |  |  |  |  |  |
| ER status |  |  |  |  | **<0.001** |
| Negative (0) | 70 (44.3) | 68 (26.6) | 121 (22.2) | 4 (9.5) |  |
| Positive (≥1%) | 88 (55.7) | 188 (73.4) | 423 (77.8) | 38 (90.5) |  |
| ER H-score |  |  |  |  | **<0.001** |
| <50 | 84 (53.2) | 98 (38.3) | 156 (28.7) | 6 (14.3) |  |
| 50-200 | 63 (39.9) | 121 (47.3) | 305 (56.1) | 17 (40.5) |  |
| >200 | 11 (6.9) | 37 (14.4) | 83 (15.2) | 19 (45.2) |  |
| ER Allred score |  |  |  |  | **<0.001** |
| 0-2 | 71 (44.9) | 70 (27.3) | 121 (22.2) | 4 (9.5) |  |
| 3-6 | 78 (49.4) | 161 (62.9) | 364 (66.9) | 24 (57.1) |  |
| 7-8 | 9 (5.7) | 25 (9.8) | 59 (10.9) | 14 (33.4) |  |
| **mRNA** |  |  |  |  |  |
| ESR1 status |  |  |  |  | **<0.001** |
| Low (<25^th^ perc) | 58 (42.0) | 56 (25.6) | 97 (20.3) | 5 (13.9) |  |
| High (>25^th^ perc) | 80 (58.0) | 163 (74.4) | 380 (79.7) | 31 (86.1) |  |
